# Identification of marginal causal relationships in gene networks from observational and interventional expression data

**DOI:** 10.1371/journal.pone.0171142

**Published:** 2017-03-16

**Authors:** Gilles Monneret, Florence Jaffrézic, Andrea Rau, Tatiana Zerjal, Grégory Nuel

**Affiliations:** 1 UMR GABI, AgroParisTech, INRA, Université Paris-Saclay, 78350 Jouy-en-Josas, France; 2 LPMA, UMR CNRS 7599, UPMC, Sorbonne Universités, 4 place Jussieu, 75005 Paris, France; Tampere University of Technology, FINLAND

## Abstract

Causal network inference is an important methodological challenge in biology as well as other areas of application. Although several causal network inference methods have been proposed in recent years, they are typically applicable for only a small number of genes, due to the large number of parameters to be estimated and the limited number of biological replicates available. In this work, we consider the specific case of transcriptomic studies made up of both observational and interventional data in which a single gene of biological interest is knocked out. We focus on a marginal causal estimation approach, based on the framework of Gaussian directed acyclic graphs, to infer causal relationships between the knocked-out gene and a large set of other genes. In a simulation study, we found that our proposed method accurately differentiates between downstream causal relationships and those that are upstream or simply associative. It also enables an estimation of the total causal effects between the gene of interest and the remaining genes. Our method performed very similarly to a classical differential analysis for experiments with a relatively large number of biological replicates, but has the advantage of providing a formal causal interpretation. Our proposed marginal causal approach is computationally efficient and may be applied to several thousands of genes simultaneously. In addition, it may help highlight subsets of genes of interest for a more thorough subsequent causal network inference. The method is implemented in an R package called MarginalCausality (available on GitHub).

## Introduction

Causal network inference is of great interest in systems biology, particularly for transcriptomic studies that aim to identify regulatory relationships among genes, i.e., gene regulatory networks. In the context of probabilistic graphical models, several algorithms have been proposed to infer the skeleton of directed, undirected, or partially-directed graphs using conditional independence tests [1, 2], score-based procedures [3–6] or mutual information [7–10]. These skeletons correspond to an equivalence class, i.e. an indistinguishable subset of graphs. Undirected graphs can be used to obtain a supergraph of the skeleton of a directed graph, which is a good starting point to infer causality when the underlying graph is unknown. Several undirected network inference methods, based on the parsimonious estimation of the inverse covariance matrix, have also been proposed for Gaussian graphical models [11, 12]. Although methods based on mutual information can also be used to infer the full graph of undirected networks [13, 14], estimating causal networks with these algorithms tends to be very computationally demanding and applicable only for low-dimensional networks. In addition, such approaches require a significant amount of interventional data to reduce the space of equivalent networks [15]. However, even with a sufficient amount of interventional data, i.e. roughly one knock-out for each gene, a directed acyclic graph (DAG) cannot generally be accurately estimated [16], perhaps due to the heterogeneous coverage of the gene network space [17]. As such, in this work we focus on estimating a few causal effects rather than attempting to infer the full network [18].

In order to reduce the complexity of the parameter search space, a topological ordering of nodes in the graph can be estimated instead of an exact network. As shown by Rau *et al.* [19], a rich set of interventional data allows the node ordering associated with a DAG to be identified. In many transcriptomic experiments, however, only a small number of interventions are available; in this work, we consider the specific case of a knock-out intervention being performed on a single gene of interest. In such a case, only a restricted equivalence class is identifiable [20], and it is reasonable to instead consider a marginal approach to estimate only the causal effects of the knocked-out gene of interest on another set of genes.

To this end, we propose a method to identify downstream causal relationships between a knocked-out gene and all other genes from replicated observational (steady state) transcriptomic data arising from an unknown graph. We first present a brief introduction to graphical models, which we use to define our model and hypothesis. The use of a mathematical operator to describe the intervention process, as defined by Pearl [21], allows the idea of causality to be formally defined in the model. This enables a closed-form expression of the likelihood to be obtained. We then illustrate the interest of our method on a set of simulated data, and we apply it to a set of microarray data from chickens carrying a functional knock-out of the growth hormone receptor gene [22]. The main advantages of the proposed marginal causal approach are that 1) it enables the accurate differentiation of downstream causal relationships from those that are upstream or simply associative; 2) it is computationally efficient, and thus simultaneously applicable to several thousands of genes; and 3) it provides a formal framework for causal interpetation. The proposed method is implemented in an R package called MarginalCausality, freely available on GitHub.

## Materials and methods

### Gaussian causal models

A directed graph G is a set of nodes ***V*** and edges E. For (X,Y)∈E, *X* is said to be a parent of *Y* (*X* ∈ pa(*Y*)), or *Y* a child of *X*, if an edge starts at *X* and points to *Y*. A directed path is a succession of nodes such that each element is a parent of the following node. A graph is said to be acyclic if there is no directed path from a node back to itself. It is then called a DAG.

A probability density *P* can be associated with a DAG. Assuming that all variables are Gaussian, such that the joint probability is a multivariate Gaussian distribution, the following factorization holds for the joint density of the graph:

$$P\left(V\right)=\prod_{V\in V}f\left(V|\text{pa}\left(V\right)\right).$$

Two graphs are said to be Markov equivalent if they have the same joint probability distribution; in this case, they belong to the same equivalence class. The mathematical do(*X* = *x*) operator [21] can be graphically defined by deleting all edges pointing from pa(*X*) to *X*. For the associated probability distribution, this corresponds to replacing the conditional distribution *f* (*X*| pa(*X*)) by **1**_*X* = *x*_.

### Model selection

**Fig 1:**
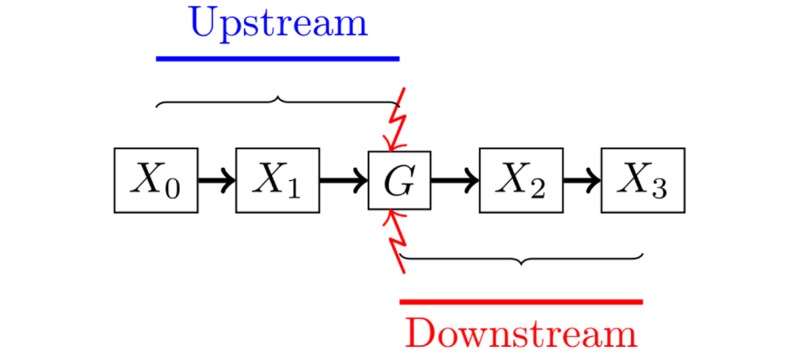
Illustration of upstream and downstream causality. Nodes *X*_0_ and *X*_1_ are both upstream causally related to knocked-out gene *G*, while nodes *X*_2_ and *X*_3_ are both downstream causally related to *G*.

Consider a graph G where an intervention was performed on a single node of interest *G*. Two kinds of causality can be defined: 1) upstream causality, which refers to edges pointing to *G*; and 2) downstream causality, which refers to edges pointing away from *G*. With the do operator defined above, when an intervention is performed on a single node *G*, it is possible to identify genes with a downstream causal relationship to *G*, i.e. the causal effects of *G* on all of the other nodes in the network (see Fig 1 for an illustration of upstream and downstream causal relationships).

Our goal is to identify genes with downstream causal relationships to a knocked-out gene of interest when the underlying graph is unknown. It is well known that when observational data alone are available, only an equivalence class for the DAG is identifiable [21]. With the addition of interventions, it is possible to reduce this set of equivalence classes, but it is still often not possible to identify a unique DAG. For this reason, we consider a marginal approach to estimate only the causal downstream relationships from a single node of interest.

Using the framework of Gaussian structural equations, three possible cases may be defined for each node *X* of the graph. First, if *X* is a child of the node of interest *G*, the following equation holds:

$$M_{1}:\;X=\mu_{X}+\alpha G+\varepsilon_{X},$$

where *μ*_*X*_ is the residual mean of *X*, *α* is the total causal effect from *G* to *X*, and *ε*_*X*_ is centered Gaussian noise with variance $\sigma_{X}^{2}$. On the other hand, if *X* is a parent of *G*, the following equation can similarly be written:

$$M_{0}:\;G=\mu_{G}+\alpha X+\varepsilon_{G},$$

where *μ*_*G*_ is the residual mean of *G*, *α* is the total causal effect from *X* to *G*, and *σ*_*G*_ the residual standard deviation of *G*. Finally, if *X* is neither a child (descendant) nor a parent (ancestor) of *G*, the model can no longer be expressed in terms of a Gaussian structural equation. However, as the pair of variables *X* and *G* may still be correlated, the pair (*X*, *G*) can be considered to be a random variable following a bivariate Gaussian distribution.

**Fig 2:**
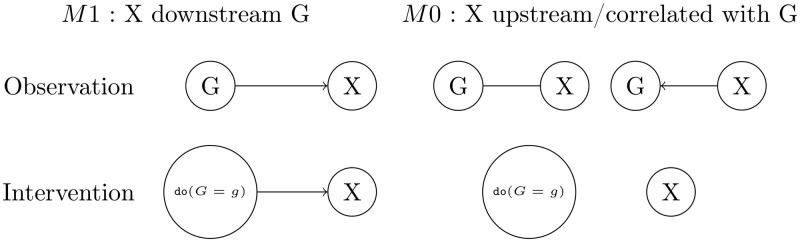
Models given observational or interventional data. Graphical representation of the *M*_1_ (downstream) and *M*_0_ (upstream or correlated) models under observational and interventional data.

We next must compute the likelihood functions associated with each of these three cases when both observational and interventional data are available. However, as illustrated in Fig 2, even with the availability of interventional data, the causal downstream model and the correlated model cannot be distinguished from one another, as their likelihoods are identical. In Model *M*_1_ (the downstream case), the distribution of *X* under the do operator is needed. In Model *M*_0_ (the upstream or correlation cases), the marginal distribution of *X* must be used. Using the Markov equivalence for observational data, all models can be reparameterized as a downstream model. Our models may thus written as follows:

$$M_{1}:Z_{1}∼N\left(\mu_{1},\sigma_{1}^{2}\right),\;Z_{2}∼N\left(\mu_{2},\sigma_{2}^{2}\right),$$

$$G=Z_{1},\;X=\alpha Z_{1}+Z_{2},$$

$$M_{0}\;:\;{\tilde{Z}}_{1}∼N\left({\tilde{\mu }}_{1},{\tilde{\sigma }}_{1}{}^{2}\right),\;{\tilde{Z}}_{2}∼N\left({\tilde{\mu }}_{2},{\tilde{\sigma }}_{2}{}^{2}\right),$$

$$G=\beta {\tilde{Z}}_{1}+{\tilde{Z}}_{2},\;X={\tilde{Z}}_{1},$$

$$M_{0}:\;\left(\begin{array}{c} G\\ X \end{array}\right)∼N\left(\left(\begin{array}{c} m_{1}\\ m_{2} \end{array}\right),\left(\begin{array}{cc} s_{1}^{2} & \rho s_{1}s_{2}\\ \rho s_{1}s_{2} & s_{2}^{2} \end{array}\right)\right).$$

We can now explicitly write the following equalities:

$$\mu_{1}=m_{1},\;\mu_{2}=m_{2}-\alpha m_{1},\;\sigma_{1}=s_{1},$$

$$\alpha =\rho s_{2}/s_{1},\;\sigma_{2}=\sqrt{s_{2}^{2}-\alpha^{2}s_{1}^{2}},$$

$${\tilde{\mu }}_{1}=m_{2},\;{\tilde{\mu }}_{2}=m_{1}-\beta m_{2},\;{\tilde{\sigma }}_{1}=s_{2},$$

$$\beta =\rho s_{1}/s_{2},\;{\tilde{\sigma }}_{2}=\sqrt{s_{1}^{2}-\beta^{2}s_{2}^{2}}.$$

We have thus obtained all of the necessary distributions to compute the respective likelihoods for each model: the joint distribution, the conditional distribution of *X* given *G*, and the marginal distribution of *X*, all with the same set of parameters. For simplification, we define {*μ*_*X*_, *σ*_*X*_} and {*μ*_*G*_, *σ*_*G*_} to be the set of residual parameters associated with *X* and *G*, respectively, and *θ* the full set of parameters. We obtain two likelihood functions, where WT (wild type) represents the observational data, and KO (knock-out) the interventional data:

$$\begin{array}{ll} l_{\text{WT}}\left(\theta \right)= & \sum_{k\in \text{WT}}\text{log}\Phi \left(X_{k}|\mu_{X}+\alpha G_{k},\sigma_{X}^{2}\right)+\text{log}\Phi \left(G_{k}|\mu_{G},\sigma_{G}^{2}\right),\\ l_{\text{KO}}^{1}\left(\theta \right)= & \sum_{k\in \text{KO}}\text{log}\Phi \left(X_{k}|\mu_{X}+\alpha G_{k},\sigma_{X}^{2}\right),\\ l_{\text{KO}}^{2}\left(\theta \right)= & \sum_{k\in \text{KO}}\text{log}\Phi \left(X_{k}|\mu_{X}+\alpha \mu_{G},\alpha^{2}\sigma_{G}^{2}+\sigma_{X}^{2}\right),\\ l_{M_{1}}\left(\theta \right)= & l_{\text{WT}}\left(\theta \right)+l_{\text{KO}}^{1}\left(\theta \right), \end{array}$$ (1)

$$l_{M_{0}}\left(\theta \right)=l_{\text{WT}}\left(\theta \right)+l_{\text{KO}}^{2}\left(\theta \right).$$ (2)

Once the likelihoods in Eqs (1) and (2) have been maximized, a Bayes factor can be calculated for each gene to choose the most probable model between *M*_0_ and *M*_1_:

$$B=\frac{P(\text{data}|M_{0})}{P(\text{data}|M_{1})}=\frac{P(M_{0}|\text{data})}{P(M_{1}|\text{data})}\times \frac{P\left(M_{1}\right)}{P\left(M_{0}\right)}.$$

The Bayes factor may then be used to order the nodes according to the strength of the downstream causal relationship with node *G*. If it is greater than 1, model *M*_0_ is preferred, whereas if it is less than 1, model *M*_1_ is preferred.

## Results

### Simulation study

In order to assess the performance of our proposed method, we performed a simulation study to ensure that it correctly distinguishes downstream causality from correlation. We considered a simulation setting similar to the experimental design of the transcriptomic data presented below. For 100 independent genes, we simulated 24 replicates in both the observational and the single-KO interventional data, using a Gaussian framework as presented in the Methods section with either a downstream causal or correlated relationship with the KO gene. For each of the 100 simulations, the Bayes factor was then calculated. Two sets of simulations were performed for each model with the same means and causal effects, but with different residual variances.

**Fig 3:**
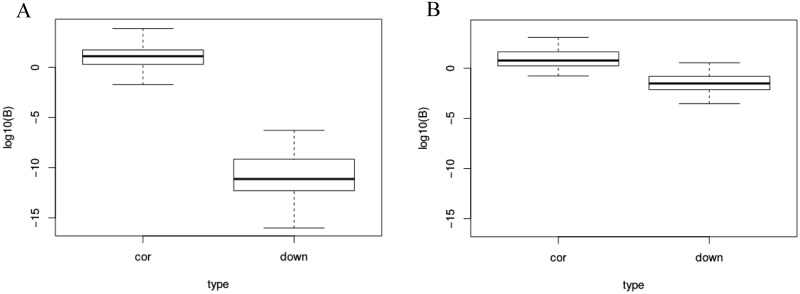
Bayes factor for the correlation (*M*_0_) and downstream (*M*_1_) models. log_10_ Bayes factor for simulated data under the downstream model (“down”) and correlation model (“cor”), with low (left; *σ*_*G*_ = 0.09, *σ*_*X*_ = 0.15) and high (right; *σ*_*G*_ = 0.3, *σ*_*X*_ = 0.5) variance.

Results for the four simulation settings are presented in Fig 3. On the left, data were simulated with *σ*_*G*_ = 0.09, *σ*_*X*_ = 0.15, and on the right, *σ*_*G*_ = 0.3, *σ*_*X*_ = 0.5. This range of values was chosen based on the transcriptomic data presented in the following section, representing small variances (within the lower quantile) and large variances (within the upper quantile), respectively. We note that for small variances, the logarithm of the Bayes factor is strongly negative for the downstream causal model, while it is around zero for the correlation model. A similar pattern is obtained for larger variances, with smaller differences between the correlation and downstream causality cases.

**Fig 4:**
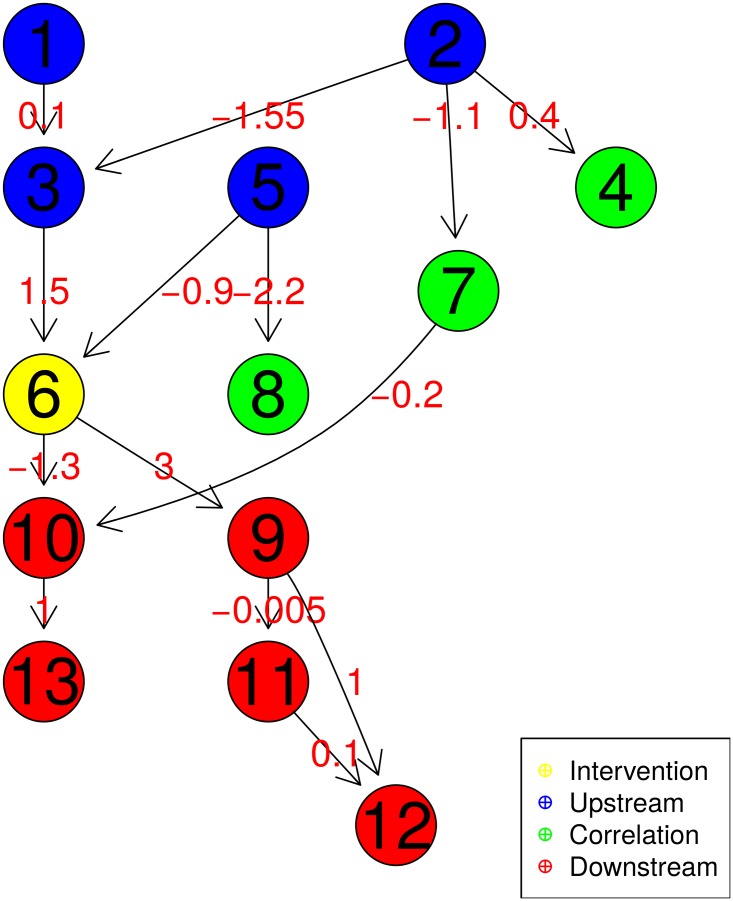
Simulated graph structure. KO interventions were simulated for Gene 6 (yellow) alone. This DAG encompasses various types of relationships with respect to the yellow node: causal upstream ancestors (blue), downstream causal genes (red), and simple correlations (green). Numbers along edges indicate the strength of direct causal effects.

In a second simulation, we investigated whether the proposed method is able to identify marginal downstream causal partners from a simulated graph. Data were simulated under a Gaussian structural equation according to the DAG structure presented in Fig 4. We simulated a knock-out intervention on Gene 6 alone; as before, 24 replicates were simulated for both the observational and interventional data for each of the other genes in the network in 100 independent runs. We note that the intervention node, represented in yellow in Fig 4, has several types of relationships with the other nodes: causal upstream ancestors (in blue), downstream causal genes (in red), and simply correlated genes (in green).

**Fig 5:**
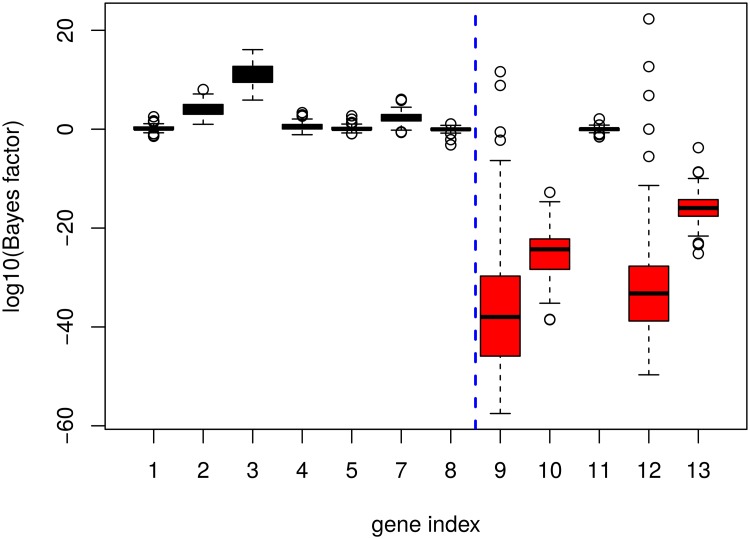
Bayes factor for the simulated graph structure. Results from 100 simulations based on the graph in Fig 4. Nodes simulated under the upstream/correlation model (*M*_0_) appear to the left in black, and those simulated under the downstream model (*M*_1_) appear to the right in red.

The values of the Bayes factor for each gene in the network are plotted in Fig 5. As before, the more strongly negative the logarithm of the Bayes factor, the more evidence there is for a downstream causal model. We note that for the genes with a truly downstream causal relationship with the KO gene (in red), the Bayes factor indeed tends to be strongly negative. Only node 11 is not detected as a descendent; this can be explained by the weak total causal effect for this node, equal to −0.015 from node 6 (the knocked-out gene) to node 11. These simulations thus confirm the ability of the proposed method to differentiate downstream causal relationships from upstream or simple correlation ones in a directed acyclic graph. An additional advantage of the marginal causal approach is that it provides an estimation of the total causal effect between the KO gene of interest and each of the others. The simulated values for these effects, as well as the estimations obtained for various numbers of replicates are provided in Table 1. The accuracy of the estimation is very robust even with a low number of replicates, with very low variability across simulations. These effects thus appear to be generally well-estimated with the proposed method.

**Table 1:** Estimated total causal effects for simulated graph structure. Mean (standard deviation) of the true and estimated total causal effects over 100 simulations for various numbers of replicates.

Gene	True value	6 WT / 6 KO	12 WT / 12 KO	24 WT / 24 KO	48 WT / 48 KO
1	0.00	0.01 (0.21)	0.04 (0.15)	0.04 (0.10)	0.02 (0.07)
2	0.00	-0.03 (0.06)	-0.04 (0.04)	-0.04 (0.02)	-0.04 (0.02)
3	0.00	0.08 (0.12)	0.10 (0.07)	0.09 (0.05)	0.09 (0.03)
4	0.00	0.00 (0.06)	-0.02 (0.04)	-0.01 (0.02)	-0.02 (0.02)
5	0.00	-0.01 (0.04)	0.00 (0.03)	-0.01 (0.02)	-0.01 (0.01)
7	0.00	0.03 (0.08)	0.05 (0.05)	0.04 (0.04)	0.04 (0.03)
8	0.00	0.04 (0.27)	-0.01 (0.18)	0.01 (0.16)	0.01 (0.10)
9	3.00	2.99 (0.17)	2.98 (0.08)	2.99 (0.23)	2.99 (0.10)
10	-1.30	-1.31 (0.08)	-1.30 (0.05)	-1.30 (0.04)	-1.31 (0.03)
11	-0.02	-0.02 (0.06)	-0.02 (0.04)	-0.01 (0.03)	-0.02 (0.02)
12	3.00	3.00 (0.11)	2.97 (0.24)	3.00 (0.14)	3.00 (0.04)
13	-1.30	-1.32 (0.16)	-1.29 (0.09)	-1.30 (0.10)	-1.31 (0.05)

**Fig 6:**
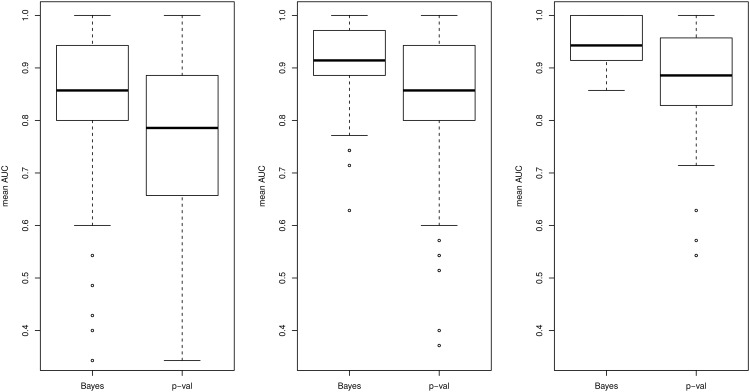
AUC for the simulated graph structure. Results from 100 simulations under three settings (5 WT / 5 KO, 10 WT / 10 KO, 25 WT / 25 KO) based on the graph in Fig 4. “Bayes” (left) corresponds to the causal marginal method, and “p-val” (right) to the *p*-values obtained using limma.

In order to compare the results of our marginal causal approach with another marginal (but non-causal) approach routinely used in practice for comparisons between two groups (here, observational and interventional), we also performed a classical differential analysis on the same set of simulated data using the R/Bioconductor limma package [23]. Briefly, limma makes use of a robust moderated two-sample t-test between the observational and interventional samples for each gene, where an empirical Bayes method is used to shrink per-gene sample variances towards a common value. We calculated the area under the receiver operating characteristic (ROC) curve (AUC) over 100 simulations to compare the sensitivity and specificity of the marginal causal approach and the differential analysis to detect downstream causal relationships. Results are presented in Fig 6. We note that for a relatively large number of replicates (10 WT / 10 KO or 25 WT / 25 KO), both methods perform very similarly, with slightly better results for the proposed causal approach. In the simulation setting with a small number of replicates (5 WT / 5 WO), the performance of the differential analysis tends to deteriorate more strongly than that of the causal approach; in particular, the AUC values much lower than in the other settings, with a large variability across the 100 simulations.

Finally, although our approach focuses on marginal causal effects and not the full network, it is of interest to compare it to a more global network-wide approach. As an illustration, we make use of the Greedy Interventional Equivalence Search (GIES) algorithm, a score-based method to infer the full directed acyclic graph based on observational and interventional data [24]. For this comparison, we focus only on the downstream causal relationships from the KO gene of interest. We use the graph structure in Fig 4 to simulate data as above (100 datasets, with 24 replicates in each of the WT and KO groups), and we define an F-score to assess the performance of each algorithm:

$$\begin{array}{c} \text{R}=\frac{\text{TP}}{\text{TP}\;\text{+}\;\text{FN}}\\ \text{P}=\frac{\text{TP}}{\text{TP}\;\text{+}\;\text{FP}}\\ \text{F-score}=2\frac{\text{R}+\text{P}}{\text{RP}} \end{array},$$

where “TP” corresponds to nodes that were simulated to be downstream of the KO gene and were correctly identified by a given method, “FP” corresponds to nodes that were not simulated to be downstream of the KO gene but were incorrectly identified by a given method, and “FN” corresponds to nodes that were simulated to be correlated/upstream of the KO gene but were incorrectly missed by a given method.

**Fig 7:**
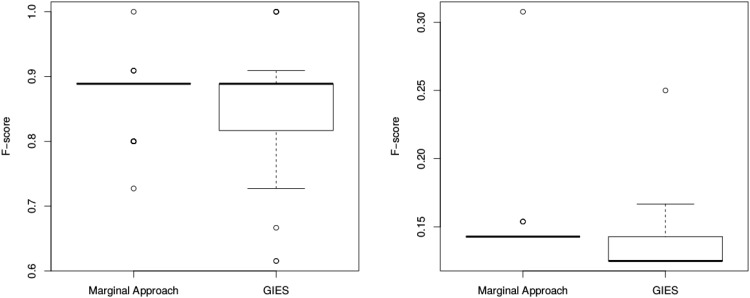
F-score of the marginal causal approach and GIES algorithm. The F-score is based on downstream (left) or not-downstream (right) links. For the marginal approach, a hard threshold of -0.5 is used for the log_10_ Bayes factor to select between models. For the GIES algorithm, the inferred topology is used to classify nodes as downstream or not.

The boxplot of F-scores for each method is shown in Fig 7, as well as the same simulation for upstream/correlation links. As our marginal approach focuses on the downstream links and does not try to infer the topology of a full network, it obtains more consistent results than the GIES algorithm. This suggests that in cases where interest is on the downstream causal links with a single KO gene, attempting to infer a complete network topology may lead to more inaccurate results than focusing on marginal causal effects.

### Real data analysis

We applied our marginal causal method to a set of transcriptomic data in the context of gene regulatory networks [25]. These data were produced at the French National Institute for Agricultural Research (INRA), in a study investigating gene expression differences between wild-type chickens and their siblings carrying a functionally inactive growth hormone receptor (GHR) gene, leading to a dwarf phenotype; in this case, a functional KO refers to a mutation for which the associated protein is generated but can no longer fulfill its role [22]. We considered the mutation of the GHR gene to be an experimental knock-out, and the expression level of the GHR gene was set to a value close to zero for the dwarf chickens. Customized Agilent microarrays were used to measure gene expression from liver samples taken from 24 wild type and 24 knock-out chickens for 18,855 genes. These data are available at GEO under the GEO accession number GSE91084.

**Fig 8:**
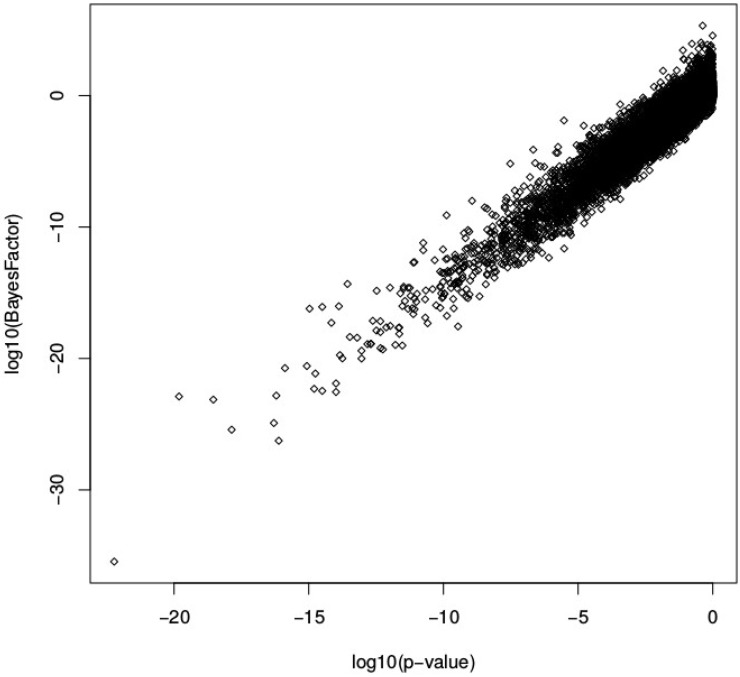
Comparison between the differential analysis and the marginal causal approach on chicken microarray data. Each point corresponds to a gene for which the differential and marginal causal analyses have been applied.

**Fig 9:**
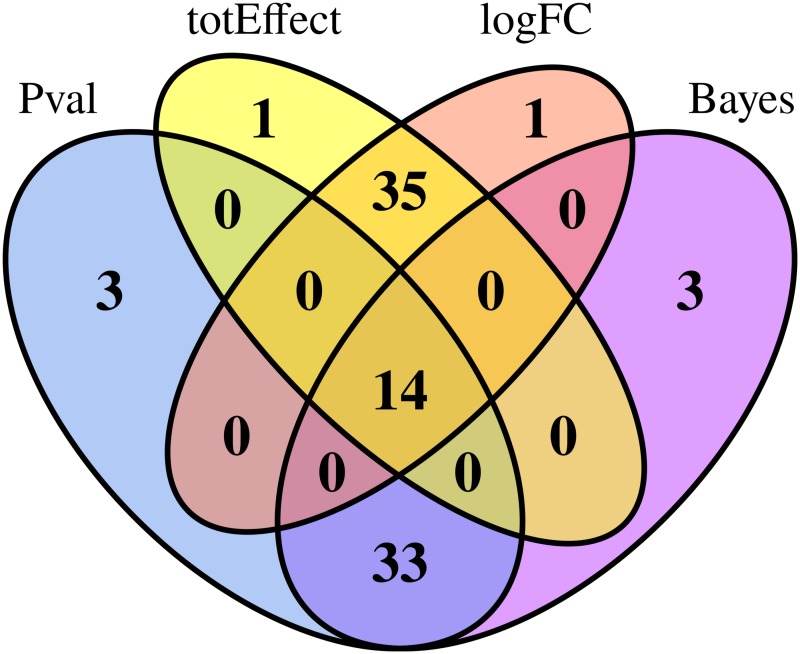
Venn diagram for the top 50 genes ranked according to the differential analysis (p-value or log-fold change) and marginal causal approach (Bayes factor or total causal effects. Ranking was performed from lowest to highest for p-values and highest to lowest for absolute total effect, absolute log fold-change, and Bayes factor.

After standard preprocessing and normalization steps, we aimed to identify genes that are downstream causally related to the GHR gene using the marginal causal method presented in this work. A classical per-gene differential analysis was also performed between wild-type and dwarf chickens using limma [23]. Fig 8 compares the Bayes factors obtained with the marginal causal method and the *p*-values of the differential analysis. In this case, the results are very similar for the two analyses, with a clear linear trend in the scatter plot. This follows the results obtained in the simulation study for more than 10 biological replicates; the added value provided by the marginal causal method, however, is that it provides a formal interpretation of the differential analysis in terms of downstream causal relationships. A similar result may be seen in Fig 9, which shows a clear correspondence between the fifty most highly ranked genes according to the Bayes Factor and *p*-values of the differential analysis. Interestingly, it also illustrates the similarity in using the estimated total causal effects and the log fold-change values (both in absolute value) to rank genes that are downstream causally related to the GHR gene.

## Discussion

We have proposed a novel approach to detect marginal causal relationships in high dimensional data when interventions are available for a single node of interest. This method was developed in the context of transcriptomic data, and can be particularly useful to perform a pre-selection of genes prior to a more thorough causal network inference. It is computationally efficient and can be simultaneously applied thousands of genes. In addition, our simulation study illustrated that the proposed method was able to accurately classify between downstream causal relationships and upstream or simple correlation relationships when the underlying DAG is unknown.

We showed that the results of differential analyses comparing KO to WT samples can indeed be interpreted as causal, given their similarity to the causal Gaussian Bayesian network. It is true that the new approach described here provides little or no improvement over classical differential analysis hypothesis tests. However, it is precisely through the new causal interpretation of these classical tests that our approach shows promise. For example, with the development of CRISPR/Cas9 genome editing [26], it is clear that the number of intervention experiments in molecular biology will increase dramatically in the coming years. In this context, although differential analysis is clearly a natural way to deal with fully controlled experiments (like randomized clinical trials), it is not particularly well-adapted for analyzing multi-factorial experiments and/or partially complete intervention designs. For these more complex types of studies, we believe that a test based on causal Gaussian Bayesian networks will be an innovative and efficient way to test and infer causality.

The proposed method relies on structural Gaussian equations, which assume linear relationships and graph acyclicity. Though not always biologically relevant, these assumptions are often made in causal gene network inference as they allow closed-form formulae of the likelihood functions to be obtained, which makes the proposed model very computationally efficient. It would be interesting in the future to evaluate whether results hold under less restrictive assumptions. The method presented here is defined in the context of interventions on one of the nodes of the network. It could similarly be applied to several interventions, if they are assumed independent of one another. However, if the interventions are causally linked, adjustments to the model would have to be considered. Finally, the proposed method was derived within an empirical Bayesian framework, where the maximum likelihood estimators were used. It would be interesting to investigate a fully Bayesian approach, using priors on the parameters that could include informative biological knowledge.
